# Nutritional Support Indications in Gastroesophageal Cancer Patients: From Perioperative to Palliative Systemic Therapy. A Comprehensive Review of the Last Decade

**DOI:** 10.3390/nu13082766

**Published:** 2021-08-12

**Authors:** Giulia E.G. Mulazzani, Francesca Corti, Serena Della Valle, Maria Di Bartolomeo

**Affiliations:** 1 Clinical Nutrition Unit, Department of Critical and Supportive Care, Fondazione IRCCS Istituto Nazionale dei Tumori, Via Venezian 1, 20133 Milan, Italy; giulia.mulazzani@istitutotumori.mi.it (G.E.G.M.); serena.dellavalle@istitutotumori.mi.it (S.D.V.); 2 Gastrointestinal Medical Oncology, Department of Medical Oncology, Fondazione IRCCS Istituto Nazionale dei Tumori, Via Venezian 1, 20133 Milan, Italy; francesca.corti@istitutotumori.mi.it

**Keywords:** gastric cancer, gastroesophageal junction cancer, nutritional therapy, nutritional support, nutrition, oncology

## Abstract

Gastric cancer treatments are rapidly evolving, leading to significant survival benefit. Recent evidence provided by clinical trials strongly encouraged the use of perioperative chemotherapy as standard treatment for the localized disease, whereas in the advanced disease setting, molecular characterization has improved patients’ selection for tailored therapeutic approaches, including molecular targeted therapy and immunotherapy. The role of nutritional therapy is widely recognized, with oncologic treatment’s tolerance and response being better in well-nourished patients. In this review, literature data on strategies or nutritional interventions will be critically examined, with particular regard to different treatment phases (perioperative, metastatic, and palliative settings), with the aim to draw practical indications for an adequate nutritional support of gastric cancer patients and provide an insight on future directions in nutritional strategies. We extensively analyzed the last 10 years of literature, in order to provide evidence that may fit current clinical practice both in terms of nutritional interventions and oncological treatment. Overall, 137 works were selected: 34 Randomized Clinical Trials (RCTs), 12 meta-analysis, 9 reviews, and the most relevant prospective, retrospective and cross-sectional studies in this setting. Eleven ongoing trials have been selected from clinicaltrial.gov as representative of current research. One limitation of our work lies in the heterogeneity of the described studies, in terms of sample size, study procedures, and both nutritional and clinical outcomes. Indeed, to date, there are no specific evidence-based guidelines in this fields, therefore we proposed a clinical algorithm with the aim to indicate an appropriate nutritional strategy for gastric cancer patients.

## 1. Introduction

Gastric cancer (GC) represents the fifth most common tumor and the third leading cause of cancer-related deaths globally, with over 1 million new cases and 783,000 deaths reported in 2018 [1]. Epidemiological differences in GC rates reflect regional variations in predisposing risk factors. Proximal tumors of the cardia and gastroesophageal junction, which recognize obesity, Gastro-Esophageal Reflux Disease (GERD), and Barrett’s esophagus as etiological factors, are more common in Western countries. Conversely, a higher prevalence of antral and distal tumors related to *Helicobacter pylori* infection and nitrite-containing diet is reported in Asian countries [2].

Although a steady decline in the incidence of GC has been observed in recent decades as a result of improved food preservation techniques and *Helicobacter pylori* infection treatment, clinicians should expect to observe more GC cases due to an ageing population in the future. While the implementation of population-based screening programs allowed for more frequent detection of early GC in Asian countries, screening is considered cost-ineffective in Western countries, resulting in routine diagnoses of more advanced stages [3].

Even though surgery remains the main curative approach for GC, 5-year survival rates are only about 50% for patients diagnosed with stage II and 20% for patients with stage III GC receiving surgery alone [4]. The introduction of multimodal treatments integrating perioperative chemotherapy or chemoradiation has led to a consistent improvement in R0 resection rates and survival outcomes, establishing integrated approaches as the standard of care for localized disease [5]. In the advanced and metastatic settings, incremental gains in overall prognosis have been achieved thanks to a deeper understanding of GC genomics and molecular profiling, leading to the introduction of targeted agents and immunotherapy in the therapeutic armamentarium [2].

Malnutrition is a severe and epidemiologically relevant cause of increased morbidity in GC patients. Proper detection and management of nutritional disorders in GC may contribute to improve quality of life (QoL), therapeutic adherence, and survival. However, to date, no definite recommendations with a high level of evidence are available in this setting [6,7].

The aim of this comprehensive review is to address the available evidence concerning nutritional support in gastroesophageal cancer patients receiving oncological treatments and to point out the importance of collaboration between the oncologist and nutritionist during the entire therapeutic process. The impact of nutritional disorders on GC treatment and prognosis will be thoroughly explored. Indications and strategies for nutritional interventions will be examined with particular regard to different treatment phases (perioperative, metastatic, and palliative settings). Lastly, an expert-opinion-based algorithm will be presented, with the aim to provide a practical tool to support therapeutic choices in everyday clinical practice.

## 2. Malnutrition: Definition, Screening Methods, Diagnosis, Impact on GC Treatment and Prognosis

According to the Global Leadership Initiative on Malnutrition (GLIM) 2018, malnutrition is defined as a clinical condition resulting from the lack of nutrient intake/assimilation, leading to weight loss and/or altered body composition and impaired clinical outcome [8]. Malnutrition in oncology ranges from 30 to 70% and is strictly connected to sarcopenia, that affects at least 50% of hospitalized cancer patients and 30% of cancer outpatients. In the esophagogastric cancer (EGC) field, 48 to 85% of patients suffer from malnutrition and sarcopenia [9,10]. The reason for the high prevalence of malnutrition in this setting is multifactorial: Tumor site (causing stenosis with partial or complete gastrointestinal tract obstruction and early satiety), operative trauma, peri-operative diet control with caloric restriction, and side effects of systemic therapies [11,12]. Malnutrition, in turn, deeply affects treatment outcomes: It reduces overall survival (OS), progression-free survival (PFS), treatment tolerance, and increases the frequency and severity of post-operative complications and treatment-related adverse events [13,14]. It is a vicious circle: Cancer increases the risk of malnutrition, whereas malnutrition enhances the risk of treatment side effects, possibly resulting in treatment discontinuation with poorer outcomes [7,10].

International guidelines of the European Society for Clinical Nutrition and Metabolism (ESPEN) and Academy of Nutrition and Dietetics and American Society for Parenteral and Enteral Nutrition (ASPEN) recommend to screen, assess, and early treat malnutrition in all oncological patients. However, as recently reported by Aprile et al. [9], only 30–70% of patients at risk of malnutrition are addressed by a nutritional assessment, and only 50% of them receive an appropriate intervention. Moreover, in the EGC field, few data exist on the actual impact of dietetic interventions and nutritional care in current clinical practice [10].

ESPEN recommends different screening tools for different settings: The adult Malnutrition Universal Screening Tool (MUST) for community outpatients, the Nutritional Risk Screening 2002 (NRS 2002) for hospitalized patients, the Mini Nutritional Assessment (MNA) for baseline evaluations of senior cancer patients, and the Patient Generated Subjective Global Assessment (PG-SGA) for cancer patients in general. As far as the diagnosis of malnutrition is concerned, GLIM has recently proposed a consensus [8].

ESPEN recommendations for cancer patients [15], recently reviewed in practical guidelines [16], suggest the following steps:

- An initial screening, preferably performed by the oncologist at the first visit.
- In case of malnutrition risk, assessment by a nutrition specialist.
- Early and personalized nutritional intervention, if indicated.
- Regular follow-up of to monitor intervention efficacy.

Different instruments for nutritional intervention are available: Dietetic counselling to promote a personalized diet, with the possible integration of oral nutritional supplements (ONS), enteral nutrition (EN), or parenteral nutrition (PN) [10]. The choice is related to the patient’s nutritional status, the integrity of the gastrointestinal tract, the type of treatment, the expected side effects, and the prognosis. No standardization exists about the time of reassessment: Monthly reassessment is advised, but the patient’s clinical condition and treatment plan must be considered. [9]

## 3. Material and Methods

We conducted a comprehensive search on Medline (PubMed) using the following keywords: “gastric cancer”, “esophagogastric cancer”, “gastroesophageal cancer”, “gastroesophageal junction cancer”, “esophagogastric junction cancer”, “cardiac cancer”, combined through AND with “nutritional therapy” or “nutrition” in search strings. We extensively analyzed the last 10 years of literature, in order to provide evidence that may fit current clinical practice both in terms of nutritional interventions and oncological treatment. Therefore, the time interval identified for the research was from January 1st 2010 to the last update made on February 18th 2021. Other relevant studies written before 2010 were not included as they were already analyzed in later reviews of the literature. Furthermore, the ClinicalTrials.gov website was checked using the following search words: “esophagogastric cancer”,” gastroesophageal cancer”, “gastric cancer” combined through AND with “nutritional therapy” or “nutrition” in search strings.

## 4. Results

**Figure 1:**
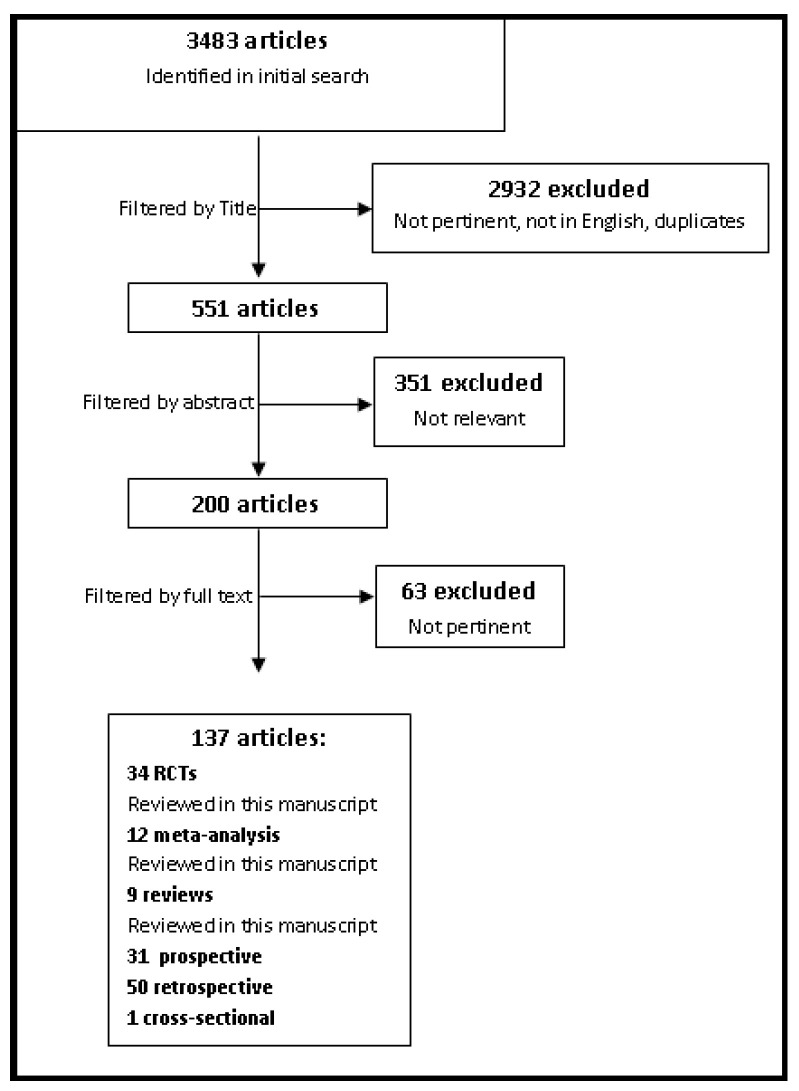
Flow-chart explaining studies selection. RCTs: Randomized Clinical Trails.

A total of 3483 articles were found in the PubMed database. A first selection was made by title and language. We therefore excluded 2550 papers as they were not pertinent or not in English. Of note, the language exclusion criterion was applied whenever the abstract of the paper was not exhaustive for the evaluation of the whole study’s content. Duplicates were eliminated and 551 articles remained. Predefined inclusion and exclusion criteria were applied, as described in Table 1, and 137 studies were selected (Figure 1).

Overall, 34 randomized clinical trials (RCTs), 12 meta-analysis, and 9 reviews were selected and analyzed in this manuscript; RCTs are listed in a comprehensive table (Table 2) for a clearer summary. Among the identified 82 prospective/retrospective/cross-sectional studies (listed in Supplementary Table S1), only the most relevant and/or significant ones were reviewed, as the vast majority of them were already included in other systematic and narrative reviews included in this manuscript.

In addition, a total of 144 ongoing trials were found on the ClinicalTrials.gov website. Of these, 124 were excluded (completed/terminated trials, duplicates, non-pertinent studies, studies dealing with different cancer types or with no specified nutritional outcomes) and 11 ongoing clinical trials were included (Table 3).

## 5. Nutritional Support Strategies

### 5.1. Perioperative Setting

Surgical resection is the only potentially curative approach for localized gastric cancer (GC). However, the 5-year overall survival (OS) rates reported do not exceed 57–70% in stage I, 32–45% in stage II, and 9–20% in stage III patients treated with surgery alone [51], highlighting the need for integrated multimodal approaches to improve patients’ outcomes.

The role of adjuvant chemotherapy was documented by the GASTRIC meta-analysis [52] and confirmed by two Asian pivotal studies. In the first Asian study, postoperative chemotherapy with S-1 following D2 nodal dissection showed OS and relapse free survival benefit compared to surgery alone in Japanese patients with stage II or III GC [53]. A second Asian study, the phase III CLASSIC trial [54], investigated the association of capecitabine plus oxaliplatin as postoperative treatment in patients with stage II-IIIB GC who received curative D2 gastrectomy. However, adjuvant chemotherapy is not always well tolerated, and less than half of the patients received the planned protocol [55,56].

The administration of preoperative chemotherapy yields several advantages over adjuvant treatment alone, including tumor downstaging or downsizing, increased rates of curative (R0) resection, potential eradication of early microscopic spread, and in-vivo assessment of drug activity [2]. The landmark MAGIC trial was the first study to assess the superiority of an integrated management encompassing surgery plus perioperative chemotherapy compared to surgery alone in patients with stage II/III esophagogastric adenocarcinoma [57]. The trial randomized 503 patients with resectable adenocarcinoma of the stomach, esophagogastric junction, or lower esophagus to either perioperative chemotherapy with epirubicin, cisplatin, and 5-fluorouracil (ECF—three cycles before surgery and three cycles after surgery) vs. surgery alone. Patients receiving perioperative ECF exhibited significantly improved disease-free survival (DFS) (Hazard ratio [HR] 0.66; 95% confidence interval [CI], 0.53–0.81; *p* < 0.001) compared to the control group, with a 13% absolute gain in 5-year OS (36% vs. 23%; HR 0.75; 95% CI, 0.60–0.93; *p* = 0.009). Moreover, it is noteworthy that only 41.6% of the patients received post-operative chemotherapy.

The French FNCLCC/FFCD phase III trial randomized 224 patients with resectable gastroesophageal adenocarcinoma to either perioperative chemotherapy with cisplatin and fluorouracil vs. surgery alone, achieving significantly improved curative resection rate (84% vs. 73%; *p* = 0.04), 5-years DFS (34% vs. 19%; HR, 0.65; 95% CI, 0.48 to 0.89; *p* = 0.003) and OS rates (38% vs. 24%; HR 0.69; 95% CI, 0.50–0.95; *p* = 0.02) in the perioperative chemotherapy arm compared to the surgery alone arm [58].

More recently, the practice-changing randomized FLOT4-AIO phase III trial established the superiority of the FLOT regimen (5-fluorouracil, folinic acid, oxaliplatin, and docetaxel) administered perioperatively (four cycles before surgery and four cycles after surgery), over ECF or ECX (epirubicin, cisplatin, and capecitabine) in patients with ≥ cT2 and/or cN+ resectable GC [59]. FLOT resulted in higher proportion of surgical resections (94% vs. 87%; *p* = 0.001), pathological early-stage tumors and R0 resections (85% vs. 78%; *p* = 0.0162), as well as increased DFS (HR 0.75; 95% CI, 0.62–0.91; *p* = 0.0036) and OS (median OS 50 months vs. 35 months; HR 0.77, 95% CI 0.63–0.94; *p* = 0.012) compared to ECF/ECX. Based on these results, FLOT represents the new standard of care for the perioperative treatment of patients with locally advanced GC who can tolerate a three-drug combination regimen, establishing perioperative chemotherapy as the standard of care in patients with ≥ stage IB disease [2,5,60].

#### 5.1.1. The Impact of Malnutrition and Nutritional Interventions in the Perioperative Setting

No data concerning patients’ nutritional status and its impact on perioperative treatment outcomes were reported in the aforementioned pivotal trials [53,54,57,58,59]. Indeed, about 30% of patients do not receive any adjuvant therapy for different reasons, mainly because of post-surgical deterioration of nutritional status [56]. Perioperative therapies may be associated with severe side effects, including anorexia, taste and smell alterations, nausea, vomiting, and diarrhoea, which in turn may exert a negative impact on the clinical and nutritional status of the patient [6]. Patients who are already malnourished before treatment start are at higher risk for more severe side effects. Notably, about 56% of patients present with clinically significant weight loss at diagnosis, and some studies demonstrated that after gastrectomy, patients typically lose 10–20% of their preoperative body weight [61]. It has been reported that patients with body weight loss received significantly less chemotherapy and developed greater toxicity [61].

Despite the advantages linked to neoadjuvant chemotherapy, a significant association exists between preoperative treatment and the increased prevalence of sarcopenia, that is associated with dose-limiting toxicities, early termination of neoadjuvant chemotherapy, and higher risk for perioperative complications [11,62,63,64]. In patients undergoing curative resection, a poor nutritional status with sarcopenia is associated with a significantly worse OS: In the study by Kamitani and colleagues [65], for example, the 3-year OS rate was 68.9% in the low skeletal muscle loss (SML) group, and 0% in the high SML group (*p* < 0.001). This is related to the impact of malnutrition on postoperative complications and mortality, because of immunological inefficiency and non-cancer-related deaths (mainly infection) [12,66,67].

Adjuvant chemotherapy should be initiated within 4–8 weeks after resection [68]. However, poor nutritional status could be a limiting factor to a timely start of the treatment, because of an increased risk of chemotherapy-related toxicity. Postoperative sarcopenia and lean body mass loss are significant risk factors for discontinuing adjuvant chemotherapy: In the report of Park et al. [69], 50% of patients with body weight loss ≥ 15% terminated adjuvant treatment due to chemotherapy-induced adverse events; furthermore, S-1 adjuvant chemotherapy itself has been found to be an independent causal factor for loss of skeletal muscle, and GC patients with severe body weight loss may even gain no benefit at all from S-1 adjuvant treatment [11,70].

Surgery itself negatively affects patients’ nutritional status, as both total and subtotal gastrectomy are direct causes of skeletal muscle loss [71]. Open total gastrectomy yields a higher risk of post-surgical malnutrition because of longer transition periods from a liquid diet to a normal diet with an insufficient caloric intake, limited absorption of nutrients, reflux esophagitis, and dumping syndrome. Laparoscopic gastric resection, on the other side, could reduce the surgical trauma, the incidence of postoperative complications (especially pulmonary infections), may assure a faster recovery, and may become an optional surgical method for underweight patients with preoperative malnutrition [66,72,73]. Considering the frequency of exocrine pancreatic insufficiency secondary to total gastrectomy, patients should be routinely screened for steatorrhea, and a pancreatic enzyme supplementation should be administered especially in those patients who apparently meet their nutritional requirements but do not reach a body weight stabilization and nutritional parameters improvement [74,75].

Nutritional intervention in patients undergoing perioperative treatment should be aimed at preventing unintentional weight loss to reduce the risk of postoperative complications and sarcopenia, thus improving short-term survival [66,76]. Some strategies can be undertaken to improve postoperative recovery and reduce complications. First of all, there is the choice of the surgical procedure: Laparoscopic surgery is demonstrated to have a shorter hospital stay and earlier initiation of adjuvant chemotherapy than open abdominal surgery [72,73]. Furthermore, a patient-tailored nutritional support, implemented by physical activity, could be useful to maintain muscle mass, reducing the risk of weight loss and sarcopenia [11,70]. However, high heterogeneity across trials on these topics were highlighted [7,21,77].

#### 5.1.2. Dietary Counselling

We did not find studies dealing with the effect of dietary counseling in the preoperative or immediate post-operative settings in GC patients. As far as the post-discharge period is concerned, some data are reported by Kim et al. [21]. In this randomized study involving 56 patients, the efficacy of a structured intervention with multiple telephone counseling sessions and questionnaires about cognitive, functional, physical, and behavioral aspects of life after gastrectomy was explored: Results are in favor of an intensive counseling intervention after gastrectomy. Interestingly, Xie et al. [35] investigated in a randomized trial the efficacy of an educational and nutritional intervention in 144 GC patients undergoing adjuvant therapy, evaluating nutritional status and chemotherapy tolerance: Their data suggest a beneficial impact of an intensive counseling approach on compliance rate to CT (73.61% vs. 55.56%, *p* = 0.024). However, considering the systematic review of Reece [76], there is no robust evidence of the positive effects of dietary intervention on weight change and oral intake.

#### 5.1.3. ONS Intervention

There is still little evidence to support the use of ONS in patients undergoing surgery for gastrointestinal cancers preoperatively, post-operatively, and post discharge [76]. According to the 2020 meta-analysis by Rinninella et al. [7], the use of ONS from the preoperative period to 3 months after surgery has a positive impact only in reducing weight loss, while other anthropometric parameters such as triceps skin fold thickness and mid-arm circumference do not differ significantly, also because of significant inter-observer heterogeneity. In the field of ONS, a noteworthy subcategory is that of an elemental diet: As observed in the RCTs by Kimura [41] and Imamura [28], elemental nutrition could be significantly beneficial in the postoperative period after total gastrectomy (rather than in distal gastrectomy), maybe because of a different risk of malabsorption. An elemental diet seems to exert a beneficial effect in preventing mucositis during adjuvant therapy: The study by Toyomasu [47], however, is based on a small sample, which is inadequate to draw significant conclusions, and a larger cohort would be needed. Another field of interest is that of immune-enriched ONS: Four different RCTs, by Ida [29], Faber [26], Feijò [43], and Ayoama [44], explore the effect of immune-enriched ONS administration at different time points in the perioperative path, but the results are uneven [78].

#### 5.1.4. EN Intervention

A higher number of studies exist about the use of enteral nutrition, through naso-enteral tube feeding or jejunostomy, in the perioperative period, particularly in the immediate post-surgery [79,80,81]; indeed, a big percentage of studies are focused on the effect of EN and enriched EN (with omega3 or nucleotides) in the perioperative period. Data from Hur et al. [17] and by Xu et al. [48] suggest a benefit in terms of QoL and immunological parameters, rather than in nutritional parameters. According to the systematic review by Rinninella [7], EN significantly improves prealbumin (PA) and albumin (ALB) and transferrin levels on the seventh post-operative day in GC compared with PN.

Prospectively, EN administered through jejunostomy and prolonged at home (Home Enteral Nutrition), allows to meet energy requirements after major surgery, as proposed in the RTCs by Baker et al. [33] and Bowrey [27]. This suggests the possible utility of the positioning of a feeding jejunostomy, either at the staging phase or during gastrectomy, although the procedure is not free from risks and complications [82,83].

#### 5.1.5. PN Intervention

In GC patients not suitable for ONS or EN, PN is the unique option with a significant improvement in ALB and PA levels compared with rehydration. The positive impact of omega-3 enriched PN compared with a control remains debated [84].

#### 5.1.6. Exercise and Nutritional Interventions

The association between nutritional support and exercise to prevent postoperative complications has been demonstrated. Resistance exercise promotes muscle protein synthesis [85,86]; the stimulatory effect on protein synthesis exerted by exogenous amino acids (by essential amino acids and leucin especially) is enhanced by prior exercise, even in the elderly. Then, appropriate planning of preoperative habilitation combining exercise and nutritional support is suggested [11,87].

### 5.2. Metastatic Setting

In the setting of advanced unresectable or metastatic GC, chemotherapy should be offered to patients with adequate performance status and organ function, as it improves QoL and survival outcomes over the best supportive care (BSC) alone [88,89]. Moreover, combination chemotherapy yields a survival advantage compared to single-agent regimens and is to be preferred over monotherapy in patients fit for combinatorial approaches [90].

First-line treatment choice is driven by tumor molecular characterization, as patients with HER2 overexpressing/amplified GC (~17–20%) benefit from the addition of trastuzumab to the platinum/fluoropyrimidine chemotherapy backbone. The randomized phase III TOGA trial evidenced an OS advantage in HER2 positive GC patients receiving trastuzumab plus chemotherapy vs. those receiving chemotherapy alone (median OS 13.8 vs. 11.1 months; HR 0.74; 95% CI 0.60–0.91; *p* = 0.0046) [91]. Based on these results, platinum-based chemotherapy plus trastuzumab followed by trastuzumab maintenance monotherapy is the recommended first-line treatment option for patients with HER2 overexpressing GC.

For patients not harboring HER2 amplification, the preferred standard initial treatment is doublet chemotherapy with platinum plus fluoropyrimidines. Cisplatin and oxaliplatin are deemed equivalent in terms of efficacy, but display a different toxicity profile, with cisplatin being associated with higher nephrotoxicity and ototoxicity rates and oxaliplatin yielding increased incidence of peripheral neuropathy and diarrhea [92]. Similarly, oral capecitabine can substitute for continuous fluorouracil infusion [93]. Irinotecan in addition to infusional 5-fluorouracil (FOLFIRI regimen) may be considered as an alternative to platinum-based first-line therapy in selected patients based on the results of a phase III clinical trial [94]. Triplets containing taxanes also represent an evidence-based fist-line therapeutic option for metastatic GC. In a phase III randomized trial [95], the addition of docetaxel to cisplatin and fluorouracil (DCF regimen) was associated with an improved response rate (37% vs. 25%, *p* = 0.01), time to progression (5.6 vs. 3.7 months; HR, 1.47 95% CI, 1.19–1.82; log-rank *p* < 0.001), and OS (9.2 vs. 8.6 months; HR, 1.29; 95% CI, 1.0–1.6; *p* = 0.02) compared to the cisplatin/fluorouracil doublet, at the price of a significant increase in overall G3-4 toxicity rate (69% vs. 59%, *p* = 0.02), including complicated neutropenia (29% vs. 12%). DCF may be therefore considered in fit, selected patients with good performance status, in whom the achievement of rapid tumor shrinkage is warranted to achieve disease and symptom control.

Second-line treatment should be offered to patients with adequate performance status, due to a proven positive impact on QoL and survival [96]. Notably, due to the rapid deterioration of clinical conditions in progressing patients, only about 30% of subjects are deemed eligible to receive second-line treatment, and about 10% are eligible to third-line therapies [6]. Second-line treatment options include chemotherapy (including paclitaxel, irinotecan, or docetaxel) [97,98,99,100], the anti-Vascular Endothelial Growth Factor Receptor 2 (VEGFR-2) agent ramucirumab as a single agent, and the combination of ramucirumab and paclitaxel [5].

Head-to-head comparison showed similar efficacy of weekly paclitaxel and irinotecan in patients progressing on first-line chemotherapy (median PFS 3.6 vs. 2.3 months, HR, 1.14; 95% CI, 0.88 to 1.49; *p* = 0.33; median OS (paclitaxel vs. irinotecan): 9.5 vs. 8.4 HR 1.13; 95% CI, 0.86 to 1.49; *p* = 0.38) [99]. Ramucirumab was shown to improve survival outcomes in comparison with a placebo in pretreated advanced GC patients in the randomized phase III REGARD trial [101]. In addition, in the phase III randomized placebo-controlled RAINBOW trial, the superiority of ramucirumab in combination with paclitaxel vs. placebo plus paclitaxel was assessed in 665 patients with advanced gastric or gastro-esophageal junction adenocarcinoma progressing on platinum-based chemotherapy [100].

A deepened insight in the molecular characterization of GC has identified four molecular types: Epstein-Barr virus (EBV), microsatellite instability (MSI), chromosomal instability, and genome stable, opening new therapeutic opportunities in defined patients’ subgroups [102]. EBV-positive and MSI-high GC are responsive to immune checkpoint inhibitors.

The efficacy of an immune-checkpoint blockade in the MSI-high GC population has been further confirmed in the subgroup analyses of two pivotal trials aimed at evaluating the activity of pembrolizumab in pretreated patients with metastatic GC and EGC, which reported overall response rates of 47–57% in the MSI-high subgroup [103,104]. More recently, a clinically significant survival benefit of pembrolizumab compared to standard first-line chemotherapy was evidenced in the MSI-high subgroup of patients enrolled in the first-line phase III KEYNOTE-062 trial (median OS not reached vs. 8.5 months, HR 0.29, 95% CI 0.11–0.81) [105]. Moreover, the Checkmate 649 phase III trial demonstrated the superiority in terms of OS (HR 0.71, 98.4% CI 0.59–0.86; *p* < 0.0001) and PFS (HR 0.68, 98% CI 0.56–0.81; *p* < 0.0001) of nivolumab in association with first-line chemotherapy compared to first-line chemotherapy alone in patients with metastatic GC and EGC with enrichment of PD-L1 expression evaluated by CPS ≥ 5 [106]. These results led to the approval of the nivolumab-plus-chemotherapy combination as an upfront treatment in this patients’ population [107].

#### The Impact of Malnutrition and Nutritional Interventions in the Metastatic Setting

Early simultaneous care with the maintenance of a good nutritional status and a better control of adverse events is crucial to improve clinical benefit in this setting [108]. As stated, malnutrition and low muscle mass at diagnosis are significantly associated with a lower OS and QoL in metastatic GC patients undergoing chemotherapy [12].

Recently, Lu et al. [109] published their data about a phase III RCT involving metastatic ECG patients undergoing first-line CT. They compared a complex and integrated early collaboration between specialists (a psychologist, dietitians, an oncologist, and an oncology nurse) with the standard of care (reactive intervention in case of specific problem), observing a significative advantage in terms of survival in the former group (median OS 14.8 vs. 11.9 months, HR 0.68, 95% CI, 0.51 to 0.9; *p* = 0.021). Furthermore, the rate of weight loss was significantly lower in the study group than in the control group (*p* = 0.032). A recent RCT by Obling et al. dealt with nutrition in the advanced gastrointestinal cancer patients setting [110]. They evaluated the longitudinal effects of supplemental Home Parenteral Nutrition (sHPN) on Fat-Free Mass (FFM), measured by the Body Impedence Assessment (BIA), in a limited sample size (47 patients), 91% of whom were undertaking palliative chemotherapy. sHPN does not increase the risk of adverse events and death and may prevent a loss of FFM with an improvement in QoL; no significant advantage in function or overall survival was identified.

An observational prospective study with a small patient sample observed that sHPN, administered even for only 1 or 2 months, is positively associated with improved QoL and nutritional status in GC patients with compromised enteral intake and malnutrition [111].

### 5.3. Palliative Setting

The American Society of Clinical Oncology (ASCO), the European Society of Medical Oncology (ESMO), and the Italian Association of Medical Oncology (AIOM) guidelines suggest that palliative care integrated early into the oncological treatment plan is helpful to optimize anticancer treatment tolerability and outcomes as well as patient’s comfort, function, and social support [112,113,114].

Since malnutrition and sarcopenia are known to be related to decreased survival and deterioration of QoL, nutritional support has also been gaining more importance in the palliative setting. A precise nutritional assessment and a good definition of oncologic programs are of utmost importance to identify the opportunity of a nutritional intervention, in a correct balance between the risks and the expected benefit on QoL and survival. According to ESPEN guidelines, we can hypothesize an advantage from nutritional therapy in the palliative setting as long as the patient is at higher risk of an earlier death due to malnutrition rather than cancer (expected prognosis 1–3 months) [115]. In this setting, nutritional care should point to favor a better QoL and alleviating symptoms.

GI symptoms (nausea, vomiting, delayed gastric emptying) may be linked to mechanical obstruction/stenosis. In this case, nutrition intervention goes together with endoscopic palliation: Duodenal stenting and gastrojejunostomy, when feasible, may be beneficial in alleviating gastric discomfort [116,117].

Although guidelines always recommend a gradual transition from the less invasive (dietary counseling and ONS) to the more invasive nutritional intervention (PN), a reduced availability of gastrointestinal tract may lead to select PN as the initial intervention, often as a supplement combined to oral food intake to meet nutritional requirements [118]. In complete or nearly complete bowel obstruction, PN is mandatory to prevent death from starvation and dehydration and could potentially offer an improvement on QoL and prolonging survival. Whenever possible, it is necessary to maintain even a minimum intake of nutrients *per os* (e.g., complex carbohydrates, liquids), with the aim to support the mucosal immune response [119].

Dietary counseling should encourage the patient to eat as tolerated, with the aim to reduce the anxiety linked to “eating something specific/healthy” or “eating enough”. For patients in the last phases of life, being pushed to eat may increase distress, with a negative impact on QoL. According to ESPEN guidelines, EN should be considered after discussion with the patient, the oncologist, and the caregivers about the prognosis, the expected benefit on QoL, and the burden associated with Home Artificial Nutrition [16,120]. EN should be considered if the gastrointestinal tract is functional, in the case of a life expectancy of several weeks or months. Endoscopic or surgical jejunostomy may be an option in the case of gastric obstruction/dysmotility, whereas a nasogastric tube or a nasojejunal tube could be considered when short-term EN is expected (usually up to 6 weeks) and/or survival is uncertain [116,117]. When EN is unfeasible or refused by the patients, according to ESPEN 2009 and 2020 guidelines [120,121], PN should be considered for patients with expected prognosis between 1 and 3 months. HPN is not recommended in the case of severe organ dysfunction or uncontrolled symptoms, Karnofsky Performance Status (KPS) < 50, short-estimated life expectancy (less than 1-3 months), and patient’s refusal. Cotogni et al. identified from a large cohort of HPN cancer patients KPS > 50, albumin level > 3.5 g/dL, and BMI > 20.5 as predictive factors for better survival [122]. Culine et al. observed a significant improvement in QoL and nutritional status in cancer patients with metastatic diseases after 4 weeks of HPN [123]. The observational study by Senesse et al. described a benefit in QoL and KPS after 1, 2, or 3 months of HPN administration, with the more relevant improvement in patients receiving HPN for 3 months [124]. More recently, Cotogni et al. confirmed in a longitudinal study that QoL, physical, role, and emotional function take advantage of HPN in advanced cancer patients [125].

### 5.4. Elderly

This population subgroup deserves specific focus. Chemotherapy benefit is also retained in the elderly population (≥70 years), even though the use of reduced doses or de-intensified schedules may be warranted to improve tolerability [91].

Data concerning the safety and efficacy of perioperative chemotherapy in the elderly population derive from subgroup analysis of the MAGIC [57] and FLOT-AIO [59] trials. In both trials, there was no clear evidence of heterogeneity of the treatment effect according to age category, thus suggesting that perioperative treatment may be equally beneficial in the elderly population. However, an upfront surgical approach may be considered in elderly patients, particularly if severe comorbidities or symptomatic disease are present [6].

In the metastatic setting, different studies and meta-analyses suggest that, in the absence of severe comorbidities or organ function impairment, the administration of systemic chemotherapy provides similar survival advantages in the elderly (i.e., age ≥ 65–70 years) compared to younger patients [126]. However, the incidence of adverse events (including neutropenia, fatigue, and infections) appears to be superior in elderly patients, suggesting that tailored and/or de-intesified treatment schedules should be considered in these population to improve tolerability and QoL without compromising oncologic outcomes. [127,128]. With regard to the choice of chemotherapeutic regimen, oxaliplatin may be preferred over cisplatin, and capecitabine may be preferred over fluorouracil in the elderly population [6].

Because of the higher incidence of comorbidities, age-related changes in pharmacokinetics and pharmacodynamics, chronic therapies and decreased organ function, elderly patients are at higher risk of toxicity, early termination of treatments, and reduced QoL. In addition, they might underreport side effects (fatigue, anorexia, pain, nausea) perceiving them as a normal part of aging, cognitive impairment, and depression. Furthermore, the physiological aging process is associated with the progressive loss of muscle mass (the amount of muscle begins to decrease after 50 years of age, and approximately 50% of the fibers are lost by 80 years of age), loss of physical function, and progressive disability. Elderly cancer patients are then more likely to present with a poorer nutritional status and a higher frailty risk at the time of diagnosis [11].

Malnutrition screening should be performed at the first oncologic evaluation: The International Society for Geriatric Oncology and ESPEN recommend the MNA Short Form, specifically designed for elderly patients. The three items on the MNA (psychological distress or acute disease in past 3 months, neuropsychological problems, and using > 3 prescription drugs) independently predicts premature discontinuation of chemotherapy. However, in a recent metanalysis [108], the Comprehensive Geriatric Assessment is suggested to guide the choice of the best nutritional support and oncologic treatment program. The timely identification of toxicities in elderly patients allow to provide appropriate assistance and improve QoL for both patient and caregiver [108]. A better control of nausea and vomiting, for example, promotes a more adequate food and liquids intake. A timely identification of reactive depression and/or anxiety, which is frequently linked to hyporexia, may help prevent further weight loss and nutritional status worsening. Prospective clinical trials specifically focused on elderly cancer patients are urgently required.

## 6. Discussion

The present research analyzed the last 10 years of literature, in order to provide evidence that may fit current clinical practice both in terms of nutritional interventions and oncological treatments. Our work thoroughly considered all setting of the disease, from perioperative treatment to the metastatic and palliative settings in order to provide a guide for all stages of patient management. One inherent limitation of this work lies in the heterogeneity of the described studies, both in terms of sample size, study procedures, and nutritional and clinical outcomes. To overcome this issue, we mainly focused on available RCTs, in order to provide the highest quality of evidence.

However, robust evidence from RCTs about the most correct nutritional approach in different phases of the disease in this specific population does not exist. The main reason lies in the ethical difficulties of conducting a well-designed study where nutritional therapy is administered only in one of two groups of malnourished patients, leaving the control group unsupported. Moreover, when patients are enrolled in clinical trials, a nutritional intervention that is different from the standard of care could become a confounding variable.

In our daily clinical practice, we often face the problem of “identifying the priority”. In GC patients, timing is everything to optimize patient’s chances, either in terms of curative therapy or benefit from first-line chemotherapy, immunotherapy, or palliative care.

Without an accurate basal clinical evaluation and a structured plan to support the patient during the oncological pathway, there is an inherent risk of worsening of the patient’s conditions. This could lead to undesired treatment reductions or interruptions, and loss of the expected benefit in terms of oncological outcomes.

The data emerging from our review show that timely nutritional support may positively impact the nutritional parameters and nutritional status of the patient, his/her quality of life, tolerance to therapies, and, consequently, treatment compliance.

As promoted in ESPEN guidelines on cancer patients (primarily addressed to oncologists, radiotherapists, and surgeons more than to nutrition specialists), a nutritional screening should be performed at the first oncologic visit, using one of the validated tools.

**Figure 2:**
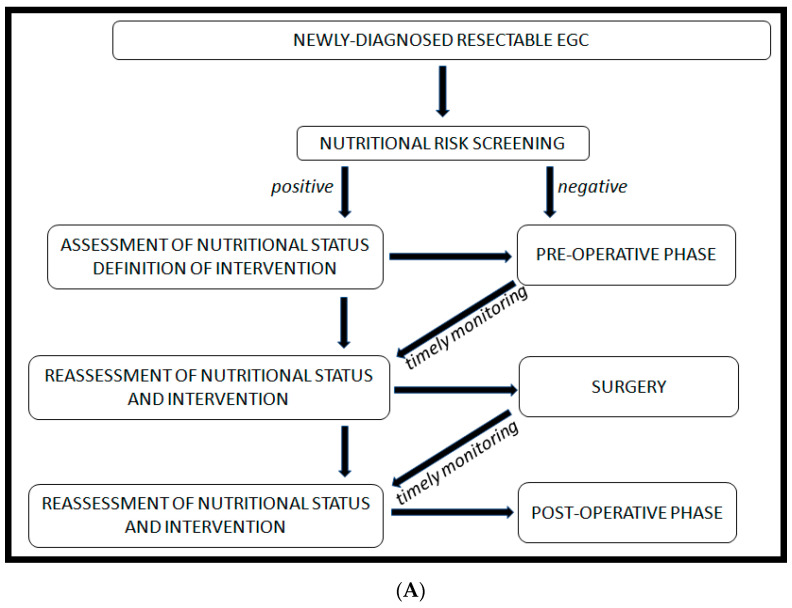

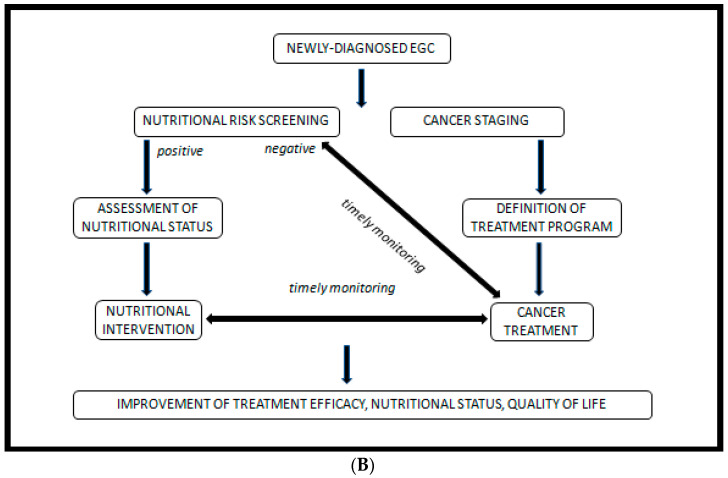
(**A**) Proposed algorithm to manage resectable esophagogastric cancer (EGC); (**B**) proposed algorithm about nutritional and oncological management of metastatic ECG patients.

In the flow-chart that we propose (Figure 2A,B), a nutritional risk evaluation must be performed at the initial phase of cancer staging and the patient should be addressed to the nutrition specialist very early in the process and regularly revaluated. In this perspective, the collaboration between nutrition specialists and oncologists is essential. Nutrition specialists, on their side, can take advantage of basal knowledge about oncologic programs, therapy plans, common drug-related side effects (especially if exacerbating gastrointestinal symptoms), for a more timely and far-sighted intervention. In this perspective, the perioperative setting takes a prominent space in our algorithm: It is the only curative setting, and it is also the one requiring the most challenging nutritional interventions.

Nutritional assessment is currently included in more research protocols because it is increasingly clear that nutritional status can significantly affect a patient’s history and adherence to treatment. Therefore, the need to better standardize nutritional interventions is unavoidable. Our proposal is a timely reassessment of the patients during oncologic staging and therapies: The re-evaluation schedule must be strictly related to the oncologic pathway in terms of treatment type, expected side effects, severity of side effects, and patient’s basal conditions. The proposed clinical outcomes could be better QoL, maintenance of a healthy nutritional status, adequate compliance to oncologic treatment, and, subsequently, longer survival. In addition, to deal with the problem of sarcopenia and the possibility to prevent or treat it, a more complex intervention should be considered, not only a nutritional support with calories, proteins, and eventually immune-enhanced nutrition, but also a structured and tailored program of physical activity. This approach would have the advantage of a higher anabolic potential.

Indeed, several RCTs are ongoing worldwide with the aim to evaluate the nutritional, immunologica, and survival outcomes of GC patients receiving different types of interventions (nutritional counselling, dietary education, physical exercise, ONS, EN, PN), in the perioperative and metastatic settings (Table 3). It is interesting to note that, among all these ongoing trials, no studies are currently analyzing the impact of microbiota modulation in the EGC population, differently from other cancer subgroups (such as melanoma). Together with the studies on immunonutrition, this field of research would be of high interest for a more personalized and more efficient nutritional support. Furthermore, other aspects of nutrition that go beyond the simple administration of macronutrients and calories, such as the association of nutrition and physical activity and psycho-social intervention, would be of great interest. Only one study concerning this trimodal therapy is ongoing at the moment.

Results of these trials are eagerly awaited to allow the implementation, in the near future, of recommendations and guidelines specifically addressing nutritional support in GC patients on the bases of high-quality and high-grade evidence.

## 7. Conclusions

In GC, a healthy nutritional status is essential for the completion of the therapeutic pathway; a continuous side-by-side teamwork between the oncologist and the nutrition specialist is unavoidable to face disease-related nutritional issues and therapy side effects, and to guarantee the maintenance of an adequate performance status. Therefore, we propose a working algorithm with the aim to optimize clinical activity. Malnourished patients or those at risk for malnutrition should be immediately referred to a nutrition specialist. However, patients with a healthy nutritional status should also be routinely re-evaluated to prevent malnutrition onset during treatments. 

## Figures and Tables

**Table 1:** Study selection criteria.

INCLUSION CRITERIA	EXCLUSION CRITERIA
Age ≥ 18 years	Age < 18 years
Gastric or esophago-gastric cancer	Mixed cancer setting (e.g., Upper GI cancers, GIT cancers)
Any oncologic treatment (surgery, chemotherapy, radiotherapy, best supportive care) at any stage	
Any nutritional intervention (counseling, oral nutritional support, enteral nutrition, parenteral nutrition)	Not a clear nutritional outcome
	Primary prevention
	Complementary/Alternative Medicine
Randomized clinical trials, prospective observational trials, case-control trials, cross-sectional trials, retrospective analysis, systematic review and metanalysis, narrative review	Case reports, case series, commentary and letters, presentation of protocol, qualitative studies

GI: Gastrointestinal; GIT: Gastrointestinal Tract.

**Table 2:** Randomized Controlled Trials evaluating nutritional interventions in gastric cancer.

Author, Year DOI	Sample Size, Region	Cancer Site, Therapy	Inclusion Criteria	Nutritional Intervention	Outcome
**Hur, 2010** [17]	54, Korea	GC, Surgery: TG, DG	Any nutritional status	Early feeding (liquid diet since 1st POD) vs. CG 6 d, 28 d FU	No differences in PO morbidity, hospitalization cost, PO pain ↓ time of gas passage ↓ readmission rate ↓ LOHS ↓ fatigue at discharge Positive effects in LOHS and aspects of QoL (secondary outcomes)
**Fujitani, 2012** [18]	244, Japan	GC, Surgery: TG	Well-nourished patients (WL of 10% or less within 6 m before surgery)	5 d of Immunonutrition PreO vs. CG	No differences in PO complications (Infections, morbidity) Negative study
**Liu, 2012** [19]	78, China	GC, Surgery: TG	Not defined (“WL recorded in all patients”)	IEEN vs. SEN vs. CG 7 d, starting 48 h PO	↑ Biochemical Nutritional parameters in IEEN and SEN vs. CG ↑ Immunological parameters in IEEN vs. SEN and CG ↓ LOHS (IEEN and EN vs. CG) No differences in PO complications Positive effect of EN on nutritional parameters; immunological effect of immune-enriched product
**Marano, 2013** [20]	109, Italy	GC, Surgery: TG	Any nutritional status (according to ESPEN Guidelines)	IEN (arginine, RNA, ω3) vs. EN (isonitrogenic, isoenergetic) trough jejunostomy Starting 6 h PO, to POD7	↓ duration of SIRS ↓ anastomotic leak and infectious complications (late PO) ↓ LOHS PO ↓ proinflammatory mediators) No differences in mortality and in nutritional parameters Partially positive
**Kim, 2014** [21]	56, Korea	GC, Surgery: TG	Any nutritional status	PPDI vs. CG 12 w, starting one d before discharge	↓ DSS ↑ KPS ↑FACT-G ↑Dietary intake ↑ ADS ↑ SDK ↑ PSS Positive effect of PPDI on nutritional outcome
**Wei, 2014** [22]	48, China	GC, Surgery: TG	BMI > 18 kg/m^2^ and <30 kg/m^2^ Severely malnourished excluded	ω3-enriched PN vs. isocaloric and isonitrogenous PN >6 consecutive d	No differences in nutritional index No PO ↑ of WBC, IL-6 and TNF-α in intervention group ↓ PO infectious complications Positive effect in flogistic parameters and PO complications
**Ding, 2015** [23]	106, China	GC, Surgery: not specified (only abstract available)	Any nutritional status	PreO 1 w EN vs. Early PO EN until 9th POD 1 w before surgery	BW, WBC, ALB, CRP, IgA, CD4, CD8, CD4/CD8, TNF-α ↑ IgG, PA in IG ↓ IL-6
**Wang, 2015** [24]	200, China	GC Surgery: TG	Any nutritional status	PreO EN (7 d before surgery) and early PO EN vs. Early PO EN 1 w before surgery for PreOP ENEN until POD 9 for both groups	↑ PA ↑ IgG level ↓ IL-6 level Positive effect of PreO EN Comment: different kinds of EN
**Li, 2015** [25]	272, China	GC Surgery: PG, DG, TG	Any nutritional status	PO EN (2nd day PO, FT) vs. isocaloric PN (1st day PO)	No differences in BW and ALB ↑ TF, PA ↓ CRP ↓ LOHS No difference in Incidence of complications Partially positive
**Faber, 2015** [26]	64, Netherlands	Esophageal or GEJ CT, RT or Surgery	Any nutritional status,	Counseling + ONS enriched in leucine, fish oil and FOS vs. Placebo oral liquid supplement if WL < 5% or Iso-caloric ONS if WL ≥ 5% 4 w before any oncologic treatment	No difference in IL-2, IFNɤ, IL-6, TNF-α ↓ PGE_2_ ↑ W ↑ ECOG PS No difference in PA, ALB Partially positive (immune function as primary outcome, sample size not adequate) Comment: Oral intake not recorded
**Bowrey, 2015** [27]	54, UK	EC Surgery: Esophagectomy or TG with placement of feeding jejunostomy tube	Any nutritional status	Overnight jejunostomy EN for 6 w after discharge (50% of energy needs supplied) vs. CG (discharge without EN, dietetic counseling. Start hEN if WL > 5% from baseline or ↓oral intake < 33% or ↓functional status) 6 w	Mean value of MAC, MAMC, TST, Handgrip > than in CG CG lost 3.9 kg (mean) more than IG Positive, the intervention is feasible, safe, acceptable Comment: 33% of patients in the CG required home EN because of clinical needs (WL, ↓ functional status)
**Imamura, 2016** [28]	112, (Japan)	GC, Surgery: DG or TG	Any nutritional status	ED group (SD + elemental diet 300 mL die) vs. CG 6–8 w (before starting AT)	↓ %BWL in TG (*p* = 0.012) not in DG (*p* = 0.059) Positive in TG
**Ida, 2017** [29]	126, Japan	GC, Surgery: TG	Any nutritional status	SD + eicosapentaenoic acid enriched ONS vs. SD From 7 to 1 d before surgery and for 21 d PO (when oral intake restarted)	No significant difference in BWL, PO complications, nutritional parameters (ALB, CRP) Negative Comment: 54% compliance PO; not evaluated total intake
**Klek, 2017** [30]	145, Poland	GC, Surgery: TG	Any nutritional status	EEN (arginine, glutammine, omega3) vs. SD 7 d, starting 12 h PO	No differences in 5-y OS ↓ risk of dying in the early period (6 m) PO in stage IV cancer Negative
**Wang, 2017** [31]	94, China	GC, NAT (capecitabine + gemcitabine, 3 courses of 21 d)	Any nutritional status	Glutamine-enriched PN vs. standard PN (isocaloric) 3 cycles of 21 d	↓ MMP-2 MMP-9 ↑ CD3+, CD4+, CD8+, CD4+/CD8+ ↑ Ig (G, A, M) ↓ Incidence of AEs Positive Comment: no data about oral intake and grade of AEs withdrawal of CT
**Zhao, 2017** [32]	120, China	GC, Surgery: DG	Any nutritional status	FF EN vs. FE EN *vs* FEP EN Start on POD1, FT for 7 d	↓ diarrhea and intestinal disorders in FEP group No differences in biochemical nutritional index (lymphocyte count, ALB, PA, TF), LOHS Positive on GI symptoms, Negative in biochemical data and LOHS
**Baker, 2017** [33]	41, UK	EC, GC Surgery: Oesophagectomy, eosophagogastrectomy or TG. CT (almost all patients)	Any nutritional status	Planned jejunostomy hEN vs. CG EN PO up to 7 d. HEN in IG (50% of estimated requirements) until clinical improvement (mean of 75 d) *vs* no intervention in CG (rescue intervention with EN in 26%)	↑ total nutritional intake ↓ BWL ↓ functional deteriorating (hand grip strength) No differences in dietary intake (not a negative impact of EN in oral intake) Positive
**Hatao, 2017** [34]	113, Japan/Taiwan	GC, Surgery: DG or TG	Any nutritional status	ONS + SD vs. PO SD 12 w after discharge	Less %BWL PO (significant only in TG group, *p* = 0.07) No significant differences in BC, biochemical parameters, QoL Partially positive, only in TG group
**Xie, 2017** [35]	144, China	GC, Surgery: TG, DG followed by AT (oxaliplatin and capecitabine, every 21 d)	Any nutritional status	Standard educational intervention during hospitalization vs. intensive individualized intervention during entire CT course entire CT course	↑ kcal intake ↑ iron intake ↑ Hb level Stabilization of BW ↑ protein and ALB ↓ rate of CT withdrawal due to the AEs (*p* = 0.004) Positive
**Catarci, 2018** [36]	43, Italy	GC, Surgery: TG, DG	Any nutritional status (classified by INA, lymphocyte count and ALB)	PES vs. CG 12 m	No differences in BMI ↑ INA class status ↑ PA after 6 mo ↑ QOL Positive
**Scislo, 2018** [37]	115, Poland	GC, Surgery: TG or STG	Normal nutritional status, mild or moderate malnutrition	Immunomodulating EN vs. CG 8–16 h after surgery until POD6	No differences in PO morbidity ↓ PO pulmonary complications ↓ PO 60-day mortality No differences in 6-m and 1-y survival Partially positive (in surgical outcome, not in OS)
**Kong, 2018** [38]	127, Korea	GC, Surgery: DG, TG, PG, PPG	Moderately or severely malnourished (PG-SGA) or BMI < 18.5 kg/m^2^	PreO and PO ONS vs. CG 2 w PreO and 4 w PO	↑ total energy intake ↓ incidence of PO complications in IG (not significant overall, significant in PG-SGA grade C, *p* = 0.24)) No differences in nutritional biochemical parameters Partially positive
**Shimizu, 2018** [39]	243, Japan	GC, Surgery: DG or TG	Any nutritional status	Early oral feeding (POD 1) vs. CG (POD 3–4) From POD 1 to POD 7	No differences in LOHS in DG patients, ↓ LOHS in TG (but not attained the target sample size) No differences in BW, rehospitalization, SIRS incidence ↑ PO complications in DG ↑ oral energy intake Negative
**Jin, 2018** [40]	80, China	GC, Surgery: TG DG	Any nutritional status	PO PN vs. CG Starting POD 1 to POD 4–8	↑ levels of PA, ALB, Hb Improved QoL ↓Anxiety and depression ↑ CD3+, CD4+, CD4+/CD8+ Positive Comment: No info about oral intake
**Kimura, 2019** [41]	106, Japan	GC, Surgery: TG, DG (31 pts received AT, S-1)	Any nutritional status	ONS (elemental diet) vs. CG 6–8 w PO	↓ %BWL (1 y PO) only in TG subgroup No difference in nutrition-related blood parameters, except for total lymphocyte count (higher in intervention group, *p* = 0.019) ED give more benefit in TG Partially positive
**Wang**, **2019** [42]	60, China	GC, Surgery: TG	Any nutritional status except for “severe malnutrition”	ERAS protocol vs. SOC Since the day of surgery to discharge	↓ PO hospital stay,↓ hospitalization costs ↓ time to first flatus, time to removal of drainage tube ↓ time to oral intake, ↓ time to mobilization ↑ PA, ALB level on POD7 ↓ CRP, N level ↑ Ig (G,A,M) and T lyn Positive effect of ERAS application
**Feijò, 2019** [43]	68, Brazil	GC, CT	Any nutritional status	Counseling + ONS-EPA/DHA (IG) vs. isocaloric ONS (CG) 30 d	↑ W No difference in CD4 and CD8 Partially positive in weight gain and immunologic profile
**Aoyama 2019** [44]	123, Japan	GC, Surgery: TG	Any nutritional status	EPA-ONS since 7 d to 1 d PreO and 21 d PO vs. Standard care	No difference in PO complications, mean reduction of LBM at 1 and 3 months after surgery Negative
**Zong, 2019** [45]	96, China	GC, CT neoadiuvant (FOLFOX4, 2 courses)	NRS ≥ 3 “Patients with indications of nutritional support” not better specified	Only CT vs. CT + ω3 oral EN, (7 d during each course) 30 days	No variation of nutritional biochemical index Positive effect in nutritional, inflammatory and intestinal flora after surger;
**Zheng, 2019** [46]	100, China	GC, Surgery: PG	Any nutritional status	Diet + probiotic vs. Diet + placebo 3–5 d PO for up to 6–7 d	↓ leukocyte inflammation index ↑ immunity index ↑ ALB and total protein Improved microflora balance (↓ Firmicutes/Bacteroidetes) Positive
**Toyomasu, 2019** [47]	22, Japan	GC, Surgery: TG or DG, followed AT (S-1)	Any nutritional status	SD + ONS (elemental diet glutamine-enriched) vs. SD without any restriction From1 to 28 d every cycle	↓ oral mucositis ↓ median BWL ↑ cumulative S-1 continuation rates Positive
**Xu, 2020** [48]	60, China	GC, Surgery: TG	Any nutritional status	EEIN (probiotics, glutamine) via FT vs. Standard EN 7 d, starting 8 h PO	No difference in nutritional variables (biochemical and anthropometric) and PO complications and LOHS ↓ CRP at day 7 ↑ CD4+ at day 7 ↓ time to first flatus Partially positive
**Wang, 2020** [49]	113, China	GC, Surgery: TG	Any nutritional status	Functional jejunal interposition) vs. Roux-en-Y group 60 months	No differences in PO food intake ↓ QoL after 12 months ↑ WL Negative
**Meng, 2021** [50]	353, China	GC, Surgery: TG or DG+/− AT	NRS > 3	Post-discharge ONS + dietary advice vs. dietary advice only 3 m	↑ BMI ↓ Sarcopenia ↑ CT tolerance ↓ Readmission rate (not significant) ↑ QoL (fatigue, appetite component) Positive effect of post- discharge ONS with dietary advice

**Table 3:** Ongoing clinical trials assessing nutritional interventions in gastric cancer patients.

Field	Title—ID n.	Study	Sample Size Therapy	Cancer Site Setting	Intervention	Primary Outcome	Region
**NUTRITION** **SYSTEMIC THERAPY**	Early IntraVenous Administration of Nutritional Support (IVANS) in Metastatic GC Patients at Nutritional Risk Undergoing I-line CT NCT03949907	RCT Phase II	192 1-line CT	GC GEC Metastatic	Nutritional counseling alone (+/− ONS) vs. Early sPN + nutritional counseling	Early sPN + nutritional counseling ↑ survival and CT feasibility	Italy Fondazione IRCCS Policlinico San Matteo
**NUTRITION** **SYSTEMIC THERAPY** **ELDERLY**	A Nutritional Management Algorithm in Older Patients with Locally Advanced EC NCT02027948	Feasibility study Interventional Single-Group Assignment	26, >65 yrs Induction CT + CT-RT + surgery or definitive CT-RT	EC GEJC Local disease	Nutritional & functional assessments - baseline - after induction CT - post-treatment Measurements: H, W, baseline WL, MNA, dysphagia MNA category for intervention - normal nutrition - at risk for malnutrition - malnourished	Feasibility of nutritional management algorithm	US, New York Memorial Sloan Kettering Cancer Center
**NUTRITION** **SYSTEMIC THERAPY**	The Analysis of Immuno-Nutrition Index in Advanced GC Receiving Preoperative Treatments: Observational Cohort Study NCT03493880	Observational Cohort Study	Child, Adult, Elderly Neoadjuvant CT or CT-RT	GC EGC Local disease	No intervention	Perioperative treatments Immuno-nutrition Index: NLR, PLR, SII, GPS, mGPS, PI, NRS2002, PNI	China, Beijing Peking University Cancer Hospital
**NUTRITION** **SYSTEMIC THERAPY**	Anorexia in Cancer Patients: Assessment of the Gut HORmone and Cytokine Profile and Body Composition, and the Impact of Dietetic Support in Patients With Gastrointestinal Cancer NCT04791254	Observational Cohort Study	450 1-line CT or immune therapy	GC GEJC Metastatic	No intervention	Differences in patterns of pre-prandial and post-prandial plasma gut hormone and CK levels between stage-standardized anorexic and non-anorexic cancer patients and age-matched healthy controls. (Ghrelin, insulin, GLP-1, PYY, pancreatic polypeptide, GIP, Chromogranin A, CCK, IL-1, IL-6, TNF-alpha) Survival at 1 y	United Kingdom, Manchester The Christie NHS Foundation Trust
**NUTRITION** **SURGERY**	Prospective Study of the Effect of Perioperative Immunonutrition on the Immune Host Defense and the Phagocytic and Bactericidal Activity of Blood Platelets in GC Patients NCT01704664	RCT Phase II	240 Neoadjuvant CT	GC Local disease	Group I: EN (Peptisorb) Group II: EN and PN with glutamine (Dipeptiven, Omegaven) in PO period Group III: oral arginine (Cubitan) Group IV: PN with glutamine preO and PO	Phagocytic and bactericidal activity of blood platelets, lymphocytes and their subpopulations, IL-1B, -6, -23 determined before and after nutritional therapy	Poland Medical University of Bialystok
**NUTRITION** **SURGERY**	The Effect of Postoperative sPN in GC Patients Who Underwent Gastrectomy: A Multicenter Prospective RCT NCT04607057	RCT Phase II	224 Adjuvant therapy	GC Local disease	D0: fasting + crystalloid fluid POD1: water sips + crystalloid fl. POD2: SFD + crystalloid fl. POD3: SFD + sPN POD4-7: SBD + sPN vs. D0: fasting + crystalloid fl. POD1: water sips + crystalloid fl. POD2: SFD + dextrose 5% water POD3: SFD + dextrose 5% water POD4-7: SBD	Total amount of kcal during hospitalization W change for 2 m PO Favorable blood test result CT feasibility ↑ QoL ↓ infection rate ↓ mortality	Korea Seoul National University Hospital
**NUTRITION** **SURGERY**	Personalized Trimodal Prehabilitation for Gastrectomy NCT04223401	RCT Phase II	128	GC Local disease	Prehab. before elective GC surgery - Nutritional intervention - Psychol. intervention - Exercise intervention vs. No Intervention	Postoperative morbidity rate by Clavien-Dindo At 90 POD	Lithuania National Cancer Institute
**NUTRITION** **SURGERY**	A RCT of Simplified Dietary Education Versus Intensive Dietary Education on Nutritional Status After Gastrectomy NCT04798820	RCT Phase II	374	GC Local disease	- SE arm in STG group - IE arm in STG group - SE arm in TG group - IE arm in TG group	W change between the two groups after surgery at immediate PO period, at 1st,3rd, 6th, 12th, 18th PO m	Korea Samsung medical center
**NUTRITION** **SURGERY**	Impact on the Hospital Stay, of an EON Protocol Applied to GC Patients After TG: A Prospective RCT NCT03257280	RCT Phase II	84	GC Local disease	EON with ONS start 48 h after TG vs. classical PO management: - 1 week of non-oral intake and tPN - At POD 7, oral contrast to prove anastomosis function - 3 d of PO oral diet	LOHS PO	Barcelona, Spain L’Hospitalet de Llobregat
**NUTRITION** **SURGERY**	A Multi-center Pilot RCT Examining the Differences of Nutritional Status of Patients Undergoing Functional Jejunal Interposition Or Roux-en-Y After TG for GC NCT01996059	RCT Phase III	500	GC Local disease	Functional Jejunal Interposition vs. Roux-en-Y	BMI 3 m PO	China, Guangdong 6th Affiliated Hospital, Sun Yat-sen University
**NUTRITION** **SURGERY**	A Prospective Multi-center RCT to Compare Survival Rates and QoL According to Follow-up Period in Patients Who Underwent Radical Gastrectomy for Advanced GC NCT04408859	RCT	886	GC Local disease	FU every 3 m after gastrectomy (Computed tomography, Chest X-ray, and blood test) vs. FU every 6 m after gastrectomy	Survival rates QoL Nutritional status	Korea National Cancer Center, et al.

## Data Availability

Not applicable.

## Supplementary Materials

The following are available online at https://www.mdpi.com/article/10.3390/nu13082766/s1, Supplementary Table S1. Complete list of reviewed prospective/retrospective/cross-sectional studies not included in references.

## Abbreviations

**LEGEND**		
ADS: Adherence to Dietary Guideline Scale	FOS: Fructo-oligosaccharides	PES: pancreatic enzyme supplementation
AEs: Adverse Events	FT: feeding tube	PG: proximal gastrectomy
ALB: albumin	FU: follow-up	PGE_2_: prostaglandin E_2_
AT: adjuvant therapy	GC: gastric cancer	PI: Prognostic Index
BC: body composition	GEC: gastroesophageal cancer	PN: parenteral nutrition
BMI: Body Mass Index	GEJC: GE junction cancer	PNI: Prognostic Nutrition Index
BWL: Body Weight Loss	GPS: Glasgow Prognostic Score	PO: post-operative
CG: control group	H: height	POD: post-operative day
CK: cytokines	h: hour	PPDI: patient participation-based dietary intervention
CRP: C reactive protein	hEN: home EN	preO: pre-operative
CT: chemotherapy	IEN: immune-EN	PPG: pylorus-preserving gastrectomy
d: day	IEEN: immune-enhanced EN	PSS: Patient Satisfaction Scale
DG: distal gastrectomy	IFN-γ: interferon-γ	QoL: quality of life
DHA: docosahexaenoic acid	IG: intervention group	RT: radiotherapy
DSS: dietary symptom scale	Ig: immunoglobulin	SDK: Scale of Dietary Knowledge
E: esophagus	IL-2, IL-6: Interleukin-2, Interleukin-6	SEN: standard enteral nutrition
EC: esophageal cancer	INA: instant nutritional assessment	SII: Systemic Immune-Inflammation Index
ECOG-PS: Eastern Cooperative Oncology Group- Performance Scale	KPS: Karnofsky Performance Status	SIRS: systemic inflammatory response syndrome
ED: elemental diet	LBM: lean body mass	SOC: standard of care
EEIN: enteral eco-immunonutrition	LOHS: length of hospital stays	sPN: supplemental parenteral nutrition
EN: enteral nutrition	m: month	STG: subtotal gastrectomy
EON: Early Oral Nutrition	MAC: mid arm circumference	TNF-α: tumor necrosis factor-α
EPA: eicosapentanoic acid	MAMC: MA muscle circumference	TG: total gastrectomy
FACT-G: Functional Assessment Cancer Therapy- General	MMP-2, MMP-9: matrix metalloproteinase 2, matrix metalloproteinase -9	tPN: total parenteral nutrition
FE: fiber-enriched	NAT: neoadjuvant therapy	TST: triceps skin fold thickness
FEP: fiber and probiotic-enriched	NLR: Neutrophil/Lymphocyte Ratio	W: weight
FF: fiber-free	NRS: Nutritional Risk Score-2002	w: week
↓: decrease	OS: overall survival	WL: weight loss
↑: increase	PA: prealbumin	y: year
BMI: Body Mass Index	H: height	PYY: gut hormone peptide YY
CCK: cholecystokinin	IE: intensive dietary education arm	QoL: quality of life
CK: cytokines	IL-1, IL-6: interleukin-1, interleukin-6	RT: radiotherapy
CT: chemotherapy	LOHS: length of hospital stays	SBD: Soft blended diet
d: day	m: month	SE: Simplified Dietary Education
EC: esophageal cancer	mGPS: modified Glasgow Prognostic Score	SFD: Semifluid diet
EN: enteral nutrition	MNA: Mini- Nutritional Assessment	SII: Systemic Immune-Inflammation Index
EON: Early Oral Nutrition	NLR: Neutrophil-to-Lymphocyte Ratio	sPN: supplemental parenteral nutrition
FU: follow-up	ONS: oral nutritional supplement	STG: subtotal gastrectomy
GC: gastric cancer	PI: Prognostic Index	TG: total gastrectomy
GEC: gastroesophageal cancer	PNI: Prognostic Nutrition Index	TNF-α:Tumor Necrosis Factor-α
GEJC: gastroesophageal junction cancer	PLR: Platelet-to-Lymphocyte Ratio	tPN: total parenteral nutrition
GIP: gastric inhibitory polypeptide	PO: post-operative	W: weight
GLP-1: glucagon-like peptide-1	POD: post-operative day	WL: weight loss
GPS: Glasgow Prognostic Score, mGPS: modified Glasgow Prognostic Score	preO: pre-operative	Y: year
