# Supplementary material for: Nutritional Support Indications in Gastroesophageal Cancer Patients: From Perioperative to Palliative Systemic Therapy. A Comprehensive Review of the Last Decade
Source: Nutrients. 2021 Aug 12;13(8):2766. doi: 10.3390/nu13082766 (PMC8400027; doi:10.3390/nu13082766)
Supplement: Supplementary file 1 [file nutrients-13-02766-s001.zip › nutrients-1317068-supplementary.pdf]

Supplementary Table 1. Complete list of reviewed prospective/retrospective/cross-sectional studies not included in references.

Field	Study type	Author, year, DOI	Title
**SURGERY Sarcopenia**	Cross-sectional	Lidoriki, 2019 10.1016/j.clnesp.2019.08.012	Associations between skeletal muscle mass index, nutritional and functional status of patients with oesophago-gastric cancer
**SYSTEMIC THERAPY**	Prospective	Dijksterhuis, 2021 10.6004/jnccn.2020.7615	Cachexia and Dietetic Interventions in Patients With Esophagogastric Cancer
**SURGERY Sarcopenia**	Prospective	Zhang, 2021 10.1093/ajcn/nqaa305	Development and validation of nomograms for the prediction of low muscle mass and radiodensity in gastric cancer patients
**SYSTEMIC THERAPY**	Prospective	Karabulut, 2021 10.1177/1078155220987291	Does nutritional status affect treatment tolerability, chemotherapy response and survival in metastatic gastric cancer patients? Results of a prospective multicenter study in Turkey
**SYSTEMIC THERAPY**	Prospective	Ma, 2021 10.1007/s00520-020-05687-4	Supplemental home parenteral nutrition improved nutrition status with comparable quality of life in malnourished unresectable/metastatic gastric cancer receiving salvage chemotherapy
**SURGERY**	Prospective	Chen, 2020 10.1016/j.nut.2019.04.009	A comparison of four common malnutrition risk screening tools for detecting cachexia in patients with curable gastric cancer
**SURGERY Sarcopenia**	Prospective	Huang, 2020 10.7150/jca.49815	Value of Sarcopenia defined by the new EWGSOP2 consensus for the prediction of Postoperative Complications and Long-term Survival
**SYSTEMIC THERAPY sarcopenia**	Prospective	Matsuura, 2020 10.1159/000502613	Correlation between Skeletal Muscle Mass and Adverse Events of Neoadjuvant Chemotherapy in Patients with Gastric Cancer
**SURGERY**	Prospective	Veeralakshmanan, 2020 10.1016/j.amsu.2020.05.032	Nutritional deficiency post esophageal and gastric cancer surgery. A quality improvement study
**SURGERY**	Prospective	Lu, 2020 10.3748/wjg.v26.i36.5508	Effects of early oral feeding after radical total gastrectomy in gastric cancer patients
**SURGERY Sarcopenia elderly**	Prospective	Fukuta, 2019 10.1016/j.nut.2018.06.022	Impact of preoperative cachexia on postoperative length of stay in elderly patients with gastrointestinal cancer
**SURGERY**	Prospective	Fujiya, 2019 10.1245/s10434-018-6342-8	Impact of Malnutrition After Gastrectomy for Gastric Cancer on Long-Term Survival
**SURGERY**	Prospective	Oh, 2019 10.1016/j.clnu.2018.02.015	Prognostic significance of perioperative nutritional parameters in patients with gastric cancer
**SURGERY**	Prospective	Lee, 2019 10.1016/j.suronc.2019.09.008	Impact of remnant stomach volume and anastomosis on nutrition and body composition in gastric cancer patients
**SURGERY Sarcopenia**	Prospective	Chen, 2019 10.1016/j.ejso.2018.09.030	Sarcopenia is an effective prognostic indicator of postoperative outcomes in laparoscopic-assisted gastrectomy
**SURGERY**	Prospective	Sagar, 2019 10.1007/s13193-019-00930-9	Perioperative Artificial Enteral Nutrition in Malnourished Esophageal and Stomach Cancer Patients and Its Impact on Postoperative Complications
**SURGERY**	Prospective	Kim, 2019 10.1097/MD.0000000000017543	The investigation of diet recovery after distal gastrectomy
**SURGERY**	Prospective	Park, 2018 10.4174/astr.2019.96.5.250	Impact of body mass index on quality of life after distal gastrectomy for gastric cancer
**SURGERY**	Prospective	Romario, 2018 10.1007/s13304-018-0522-8	Enhanced recovery after surgery in gastric cancer which are the main achievements from the Italian experience (ERAS)
**SURGERY**	Prospective	Manfredelli, 2017 10.1245/s10434-017-5945-9	Could a Feeding Jejunostomy be Integrated into a Standardized Preoperative Management of Oeso-gastric Junction Adenocarcinoma
**SURGERY**	Prospective	Makuuchi, 2017 10.1016/j.ejso.2016.07.140	Enhanced recovery after surgery for gastric cancer and an assessment of preoperative carbohydrate loading
**SURGERY**	Prospective	Rodrigues, 2017 10.1177/0884533616653807	Nutrition and Immune-Modulatory Intervention in Surgical Patients With Gastric Cance
**SURGERY**	Prospective	Kobayashi, 2017 10.1007/s10120-016-0668-3	Multi-institutional prospective feasibility study to explore tolerability and efficacy of oral nutritional supplements for patients with gastric cancer undergoing gastrectomy
**SURGERY Sarcopenia**	Prospective	Wang, 2016 10.1245/s10434-015-4887-3	Sarcopenia Adversely Impacts Postoperative Clinical Outcomes Following Gastrectomy in Patients with Gastric Cancer: A Prospective Study
**SYSTEMIC THERAPY**	Prospective	Sunde, 2016 10.1111/dote.12352	Relief of dysphagia during neoadjuvant treatment for cancer of the esophagus or gastroesophageal junction
**SURGERY**	Prospective	Ohkura, 2016 10.1186/s12957-016-1013-3	Effectiveness of postoperative elemental diet (Elental®) in elderly patients after Gastrectomy
**SURGERY Sarcopenia elderly**	Prospective	Zhou, 2016 10.1016/j.jss.2016.12.014	Sarcopenia: a new predictor of postoperative complications for elderly gastric cancer patients who underwent radical gastrectomy
**SURGERY**	Prospective	Honda, 2015 10.1245/s10434-015-4696-8	Development and Validation of a Disease-Specific Instrument to Measure Diet-Targeted Quality of Life for Postoperative Patients with Esophagogastric Cancer
**SURGERY**	Prospective	Lee, 2015 10.4174/astr.2016.90.2.79	Effects of intensive nutrition education on nutritional status and quality of life among postgastrectomy patients
**SYSTEMIC THERAPY**	Prospective	Qui, 2015 10.1007/s00520-014-2523-6	Nutrition support can bring survival benefit to high nutrition risk gastric cancer patients who received chemotherapy
**SYSTEMIC THERAPY Palliative**	Prospective	Endo, 2014 10.1002/jso.23486	Efficacy of Endoscopic Gastroduodenal Stenting for Gastric Outlet Obstruction due to Unresectable Advanced Gastric Cancer: A Prospective Multicenter Study
**SURGERY**	Prospective	Guo, 2010 10.1111/j.1440-1746.2009.06198.x	Screening of the nutritional risk of patients with gastric carcinoma before operation by NRS 2002 and its relationship with postoperative results
**SURGERY**	Retrospective	Qian, 2021 10.1186/s12957-021-02132-6	Preoperative Controlling Nutritional Status (CONUT) score predicts short-term outcomes of patients with gastric cancer after laparoscopy-assisted radical gastrectomy
**SYSTEMIC THERAPY**	Retrospective	Watanabe, 2021 10.21873/invivo.12292	Effect of Prognostic Nutrition Index in Gastric or Gastro-oesophageal Junction Cancer Patients Undergoing Nivolumab Monotherapy
**SYSTEMIC THERAPY**	Retrospective	Fukahori, 2021 10.1007/s00520-020-05479-w	A retrospective cohort study to investigate the incidence of cancer-related weight loss during chemotherapy in gastric cancer patients
**SURGERY Sarcopenia**	Retrospective	Uchida, 2021 10.1007/s00595-020-02225-x	Association between low preoperative skeletal muscle quality and infectious complications following gastrectomy for gastric cancer
**SURGERY Sarcopenia**	Retrospective	Kouzu, 2021 10.3892/mco.2020.2165	Impact of postoperative reduced skeletal muscle on prognosis after recurrence in gastric cancer
**SYSTEMIC THERAPY**	Retrospective	Ma, 2021 10.1002/cam4.3604	Prognostic significance of nutritional markers in metastatic gastric and esophageal adenocarcinoma
**SURGERY**	Retrospective	Hsueh, 2020 10.21873/invivo.12106	Predicting Postoperative Events in Patients With Gastric Cancer A Comparison of Five Nutrition Assessment Tools
**SURGERY**	Retrospective	Lim, 2020 10.3390/nu12071905	Nutritional and Clinical Factors Affecting Weight and Fat-Free Mass Loss after Gastrectomy in Patients with Gastric Cancer
**SURGERY**	Retrospective	Lin, 2020	Which Nutritional Scoring System Is More Suitable for Evaluating the Short- or Long-Term

		10.1007/s11605-019-04360-4	Prognosis of Patients with Gastric Cancer Who Underwent Radical Gastrectomy
**SURGERY**	Retrospective	Sugavara, 2020<br>10.1002/jpen.1978	Geriatric Nutrition Index Influences Survival Outcomes in Gastric Carcinoma Patients Undergoing Radical Surgery
**SURGERY**	Retrospective	Park, 2020<br>10.1016/j.ejso.2019.10.024	Prognostic significance of body mass index and prognostic nutritional index in stage II III gastri
**SURGERY**	Retrospective	Kwon, 2020<br>10.5230/jgc.2020.20.e17	Advantages of Distal Subtotal Gastrectomy over Total Gastrectomy in the Quality of Life of Long-Term Gastric Cancer Survivors
**SURGERY**	Retrospective	Zhang, 2020<br>10.1111/jhn.12861	Marked loss of adipose tissue during neoadjuvant therapy as a predictor for poor prognosis in patients with gastric cancer: A retrospective cohort study
**SURGERY**	Retrospective	Xu, 2020<br>10.1002/jpen.1944	Impact of Preoperative Short-Term Parenteral Nutrition Support on the Clinical Outcome of Gastric Cancer Patients: A Propensity Score Matching Analysis
**SURGERY**	Retrospective	Wu, 2020<br>10.1016/j.clnu.2020.06.015	Parenteral glutamine supplementation improves serum albumin values in surgical cancer patients
**SURGERY**	Retrospective	Xue, 2020<br>10.1097/MD.0000000000019270	Evaluation and validation of the prognostic value of nutrition and immunity parameters in gastric cancer after R0 resection
**SURGERY**	Retrospective	Wang, 2020<br>10.7150/ijms.46530	postoperative BMI loss at one year correlated with poor outcomes in chinese gastric cancer patients
**SURGERY**	Retrospective	Claudino, 2020<br>10.1016/j.nut.2019.110590	Postoperative complication rate and survival of patients with gastric cancer undergoing immunonutrition: A retrospective study
**SURGERY**<br>**Sarcopenia elderly**	Retrospective	Zhang, 2020<br>10.2147/OTT.S264199	combining the fibrinogen-to-pre-albumin ratio and prognostic nutritional index (fpr-pni) predicts the survival in elderly gastric cancer patients after gastrectomy
**SURGERY**<br>**Sarcopenia**	Retrospective	Sugawara, 2020<br>10.1016/j.ejso.2020.04.044	Poor nutritional status and sarcopenia influences survival outcomes in gastric carcinoma patients undergoing radical surgery
**SURGERY**	Retrospective	Shinohara, 2020<br>10.1007/s13193-019-00999-2	Feasibility and Safety of Early Oral Feeding in Patients with Gastric Cancer After Radical Gastrectomy ERAS
**SURGERY**	Retrospective	Jang, 2020<br>10.1016/j.clnu.2020.02.019	Tolerability of early oral nutrition and factors predicting early oral nutrition failure after gastrectomy
**SURGERY**	Retrospective	Wang, 2020<br>10.1155/2020/6813176	Prealbumin-to-Globulin Ratio Can Predict the Chemotherapy Outcomes and Prognosis of Patients with Gastric Cancer Receiving First-Line Chemotherapy
**SURGERY**<br>**SYSTEMIC THERAPY**	Retrospective	Xiao, 2020<br>10.1038/s41430-019-0502-1	Association among the prognostic nutritional index, completion of adjuvant chemotherapy, and cancer-specific survival after curative resection of stage II/III gastric cancer
**SYSTEMIC THERAPY**	Retrospective	Wu, 2020<br>10.6133/apjcn.202012_29(4).0005	Nutritional statuses before and after chemotherapy predict the prognosis of Chinese patients after gastrectomy for gastric cancer
**SURGERY**	Retrospective	Kim, 2019<br>10.1007/s11605-019-04301-1	Comparison of Postoperative Nutritional Status after Distal Gastrectomy for Gastric Cancer Using Three Reconstructive Methods a Multicenter Study of over 1300 Patient
**SURGERY**<br>**Sarcopenia**	Retrospective	Koch, 2019<br>10.1371/journal.pone.0223613	Sarcopenia as a prognostic factor for survival in patients with locally advanced gastroesophageal adenocarcinoma
**SURGERY**<br>**Sarcopenia**	Retrospective	Ma, 2019<br>10.1016/j.nut.2018.10.025	Impact of sarcopenia on clinical outcomes after radical gastrectomy forpatients without nutritional risk
**SURGERY**<br>**Sarcopenia**	Retrospective	Shi, 2019<br>10.1159/000504283	Sarcopenia is Associated with PerioperativeOutcomes in Gastric Cancer Patients Undergoing Gastrectomy
**SURGERY**	Retrospective	Wang, 2019<br>10.2147/CMAR.S191333	Prognostic significance of preoperative albumin-to-globulin ratio and prognostic nutritional index combined score in Siewert type 3 adenocarcinoma of esophagogastric junction
**SURGERY**<br>**Sarcopenia**	Retrospective	Bitencourt, 2019<br>10.1590/0100-3984.2019.0009	Computed tomography-measured body composition correlation with postoperative morbidity and mortality
**SURGERY**<br>**Sarcopenia elderly**	Retrospective	Yu, 2019<br>10.5230/jgc.2019.19.e22	Bioelectrical Impedance Analysis for Prediction of Early Complications after Gastrectomy in Elderly Patients with Gastric Cancer
**SURGERY**<br>**Sarcopenia**	Retrospective	Zhang, 2018<br>10.3747/co.25.4014	Computed tomography-quantified body composition predicts short-term outcomes after gastrectomy in gastric cancer
**SURGERY**	Retrospective	Park, 2018<br>10.4174/astr.2018.95.4.192	Midterm body composition changes after open distal gastrectomy for early gastric cancer
**SURGERY**	Retrospective	Ryo, 2018<br>10.1245/s10434-018-07121-w	The Controlling Nutritional Status Score Serves as a Predictor of Short- and Long-Term Outcomes for Patients with Stage 2 or 3 Gastric Cancer
**LONG TERM OUTCOME**	Retrospective	Kim, 2017<br>10.5230/jgc.2017.17.e12	Actual 5-Year Nutritional Outcomes of Patients with Gastric Cancer
**LONG TERM OUTCOME**	Retrospective	Liu, 2017<br>10.2147/OTT.S132432	Gastric cancer, nutritional status, and outcome
**SURGERY**	Retrospective	Choi, 2017<br>10.1007/s11605-016-3297-6	Complications of Feeding Jejunostomy Tubes in Patients with Gastroesophageal Cancer
**PALLIATIVE CARE**	Retrospective	Min, 2017<br>10.4174/astr.2017.93.3.130	Laparoscopic gastrojejunostomy versus duodenal stenting in unresectable gastric cancer with gastric outlet obstruction
**SURGERY**<br>**Sarcopenia**	Retrospective	Sakurai, 2017<br>10.1245/s10434-017-5875-6	Adverse Effects of Low Preoperative Skeletal Muscle Mass in Patients Undergoing Gastrectomy for Gastric Cancer
**PALLIATIVE CARE**	Retrospective	Mimatsu, 2017<br>10.21873/anticanres.11812	Utility of Inflammatory Marker- and Nutritional Status-based Prognostic Factors for Predicting the Prognosis of Stage IV Gastric Cancer Patients Undergoing Non-curative Surgery
**SYSTEMIC THERAPY**	Retrospective	Palmela, 2017<br>10.5230/jgc.2017.17.e8	Body Composition as a Prognostic Factor of Neoadjuvant Chemotherapy Toxicity and Outcome in Patients with Locally Advanced Gastric Cancer
**SURGERY**<br>**Sarcopenia elderly**	Retrospective	Fukuda, 2016<br>10.1007/s10120-015-0546-4	Sarcopenia is associated with severe postoperative complications in elderly gastric cancer patients undergoing gastrectomy
**SURGERY**	Retrospective	Fukuda, 2015<br>10.1245/s10434-015-4820-9	Prevalence of Malnutrition Among Gastric Cancer Patients Undergoing Gastrectomy and Optimal Preoperative Nutritional Support for Preventing Surgical Site Infections
**SURGERY**	Retrospective	Sierzega, 2015<br>10.1007/s11605-014-2720-0	Feasibility and Outcomes of Early Oral Feeding After Total Gastrectomy for Cancer
**SURGERY**<br>**SYSTEMIC THERAPY**	Retrospective	Yamaoka, 2015<br>10.1007/s10120-014-0365-z	Skeletal muscle loss after total gastrectomy, exacerbated by adjuvant chemotherapy
**SURGERY**	Retrospective	Jeong, 2014<br>10.1007/s10120-013-0275-5	The safety and feasibility of early postoperative oral nutrition on the first postoperative day after gastrectomy for gastric carcinoma
**SURGERY**	Retrospective	Chen, 2014<br>10.6133/apjcn.2014.23.4.15	Early enteral nutrition after total gastrectomy for gastric cancer
**SURGERY**<br>**SYSTEMIC THERAPY**	Retrospective	Aoyama, 2013<br>10.1245/s10434-012-2776-6	Body Weight Loss After Surgery is an Independent Risk Factor for Continuation of S-1 Adjuvant Chemotherapy for Gastric Cancer
**PALLIATIVE CARE**	Retrospective	Mendelsohn, 2011<br>10.1016/j.gie.2011.01.042	Carcinomatosis is not a contraindication to enteral stenting in selected patients with malignant gastric outlet obstruction
